# A Compact Rat-Race Coupler with Harmonic Suppression for GSM Applications: Design and Implementation Using Artificial Neural Network

**DOI:** 10.3390/mi14071294

**Published:** 2023-06-24

**Authors:** Salah I. Yahya, Saeed Roshani, Mohammad Ami, Yazeed Yasin Ghadi, Muhammad Akmal Chaudhary, Sobhan Roshani

**Affiliations:** 1 Department of Communication and Computer Engineering, Cihan University-Erbil, Erbil 44001, Iraq; salah.ismaeel@koyauniversity.org; 2 Department of Software Engineering, Faculty of Engineering, Koya University, Koya KOY45, Iraq; 3 Department of Electrical Engineering, Kermanshah Branch, Islamic Azad University, Kermanshah 6718997551, Iran; s_roshany@yahoo.com (S.R.); mohamad.ami777@gmail.com (M.A.); 4 Software Engineering and Computer Science Department, Al Ain University, Al Ain 15551, United Arab Emirates; yazeed.ghadi@aau.ac.ae; 5 Department of Electrical and Computer Engineering, College of Engineering and Information Technology, Ajman University, Ajman 346, United Arab Emirates; m.akmal@ajman.ac.ae

**Keywords:** rate race coupler, resonator, neural network, filter, GSM

## Abstract

In this paper, a compact microstrip rat-race coupler at a 950 MHz operating frequency is designed, simulated, and fabricated. New branches are proposed in this design using high-/low- impedance open-ended resonators. In the conventional rat-race coupler, there are three long λ/4 branches and a 3λ/4 branch, and they occupy a very large area. In the presented designed, three compact branches are proposed for use instead of three λ/4 branches and an ultra-compact branch is suggested for use instead of the 3λ/4 branch. Additionally, an artificial neural network (ANN) approach is incorporated to improve the performance of the resonators using a radial basis function (RBF) network. The proposed compact structure has achieved a reduction of more than 82% compared with the size of the conventional coupler structures. Additionally, the proposed coupler can suppress the 2nd up to the 5th harmonic to improve the performance of the device.

## 1. Introduction

Rat-race couplers (RRCs) have been widely used in microwave circuits for many years. These couplers are essential components of many applications, including power dividers, mixers, and amplifiers, due to their excellent isolation, phase balance, and broadband operation. However, harmonic distortion can significantly degrade the performance of microwave circuits, especially when high power levels are involved. Therefore, it is necessary to design compact RRCs with harmonic suppression to improve the performance of microwave circuits. Compact rat-race couplers have attracted significant attention in recent years due to the small size they occupying microwave devices and their low cost. In particular, researchers have focused on developing microwave devices, such as compact rat-race couplers with harmonic suppression capabilities, to reduce unwanted harmonic signals generated by nonlinear devices in microwave and RF systems [1,2,3]. This literature review aims to provide an overview of recent research on compact rat-race couplers with harmonic suppression capabilities.

A common approach to achieving harmonic suppression in compact rat-race couplers and power dividers involves the introduction of different resonators [4,5] and filters [6,7]. For example, in [8], the authors proposed a balanced rat-race coupler by presenting the quad-mode resonator. The coupler could achieve a filtering response up to the second harmonic. In [9], a compact rat-race coupler with a stepped-impedance resonator (SIR) was proposed. This also provided a wide stop band and harmonic suppression. This coupler could achieve a high level of suppression for the higher harmonics.

Another approach to achieving harmonic suppression is to use novel coupler structures. For example, in [10], a compact rat-race coupler with an asymmetrical branch–line structure was proposed. The coupler achieved more than 20 dB suppression of the second, third, and fourth harmonics over a wide frequency range. In [11], a compact rat-race coupler with a modified branch–line structure and shunt open stubs was proposed. The coupler achieved a wide suppression band in the frequency response.

In addition to the compact rat-race couplers, other coupler structures have also been proposed for harmonic suppression. In [12], a compact Wilkinson power divider with harmonic suppression capabilities was proposed. This power divider achieved suppression for the second harmonic. In [13], a compact coupler with harmonic suppression capabilities was proposed using meandered lines. The coupler could achieve a suppression greater than 20 dB suppression for the second and third harmonics over a wide frequency range.

Study [14] introduced a compact rat-race coupler design with harmonic suppression using stubs and meandered lines and exhibited a wide bandwidth and high isolation. Additionally, in [15], a compact rat-race coupler with harmonic suppression was introduced using meandered lines and synthesized lines. This featured a simple and compact design and good performance. 

A rat-race coupler with harmonic suppression based on folded structures was proposed in [16] and this offered a compact size and high harmonic suppression level. Moreover, [17], proposed a compact rat-race coupler with harmonic suppression using capacitor loading. The method featured a simple and effective design and high harmonic suppression performance. In [18], a compact rat-race coupler with harmonic suppression was introduced using a ring resonator. This coupler offers 3rd harmonic suppression and a wide bandwidth. Moreover, the utilization of photonic crystals [19,20,21] can be applied for higher-frequency operations for microwave circuits [22,23,24]. 

Additionally, recently, neural network techniques have been used to improve the performance of electronic circuits [25,26,27] that have been applied in the design of BPFs and couplers [28,29,30,31]. Among the neural networks, RBF networks are useful tools to model nonlinear systems, such as microwave systems. In [32], the RBF-ANN model is used to model the small-signal behavior of the microwave transistors. Especially, the intermodulation distortion behavior of MESFETs is modeled using RBF in [32], which the results of this study show to be very accurate and efficient via an RBF-ANN approach. Moreover, it is claimed in [33] that using regular MLP in modeling nonlinear behavior of microwave devices was failed to converge to right solutions, but the RBF network improved the results to the right solution. Additionally, it is shown in [31,34,35] that the ANN approach can help to improve the microwave devices functionality and achieve the desired frequency parameters.

In addition, there are several works that have designed the RRC by combining of the aforementioned techniques. Some of these research results are explained in the following discussion. In [36], slow-wave broadside-coupled microstrip lines are used to create a compact rat-race coupler with a wide response. Benefiting from the slow-wave line and slow-wave coupled-line structure, an ultra-compact rat-race coupler with a planar structure is realized. This coupler has a 94% size reduction, compared with conventional RRC, but it offers no harmonic suppression. This coupler has a complex structure, which complicates the design process. In [37], the potential of space-filling curves in the miniaturization of hybrid couplers with fractal structures is demonstrated. This structure has been achieved through the replacement of straight transmission lines segments by space-filling curve segments with the same electrical characteristics. The reported RRC has an 87% size reduction compared with conventional RRC, but has no harmonic suppression. A microwave RRC, with a 56% size reduction and high port isolations, is introduced in [36]. Two short-circuited stubs are used in this RRC structure to reduce circuit size, but this structure also has no harmonic suppression. In [38], a compact microwave RRC with harmonic eliminations is designed. In this coupler, stub-loaded transmission lines are used instead of long conventional lines, providing an 85% size reduction compared with conventional RRC. This coupler works at 2.4 GHz and provides harmonic suppression bands up to 24 GHz, suppressing the 2nd–10th harmonics. A defected ground structure (DGS) is used to design a small RRC coupler with harmonic suppression in [39]. With this structure, a 54% size reduction is obtained and only the3rd harmonic is suppressed. However, the applied DGS structure has some drawbacks. The fabrication process for these structures can be complex and expensive, which can increase the overall cost of the device and need extra steps in the fabrication, which is undesirable [40]. In [41], C-SCMRC resonators are used in the RRC, resulting in a 55% size reduction and 2nd and 3rd harmonic suppression. The applied C-SCMRC resonators have the same electrical characteristics as conventional microstrip lines at the operating frequency, but suppress them at higher frequencies. The applied resonators in this RRC reduce size but increase insertion loss parameters, which is undesirable. A rat-race coupler with a filtering response is presented in [42]. This is based on coupled-line structures that can suppress the 2nd harmonic. Applied coupled lines in this RRC provide wideband responses with 35% fractional bandwidth and 2nd harmonic suppression. The insertion loss of this coupler is high, which is not desirable.

Overall, compact rat-race couplers with harmonic suppression capabilities have been extensively researched in recent years. Various approaches have been proposed for this task, including one involving the introduction of frequency-dependent phase shifts and use of novel coupler structures. The development of these compact couplers has significant potential for reducing the size and cost of microwave and radio frequency (RF) systems, while also maintaining a high performance.

This paper presents a novel design of a compact RRC with harmonic suppression for microwave circuits. The designed ANN model is used to predict the response of the basic presented low pass filter (LPF). Then, the dimensions of an LPF with desired output parameters are obtained by using the proposed ANN model. The presented ANN-RBF model can be used to designing and improve the performance of any microwave device structure. The output parameters of the predicted structure, such as frequency, can be predicted in the predefined range used for network training. For the wider range of output parameters, the network should be retrained, at which point it can predict the output parameters in the new range. The proposed design employs a novel approach to reduce the second, third, fourth, and fifth harmonic levels in the proposed power divider via designed filter structure in which the filter dimensions are predicted by using the presented approach of ANN modeling and RBF networks. The resulting coupler is highly compact, exhibiting excellent performance characteristics, including high isolation, low insertion loss, and harmonic suppression.

## 2. Design Procedures

**Figure 1:**
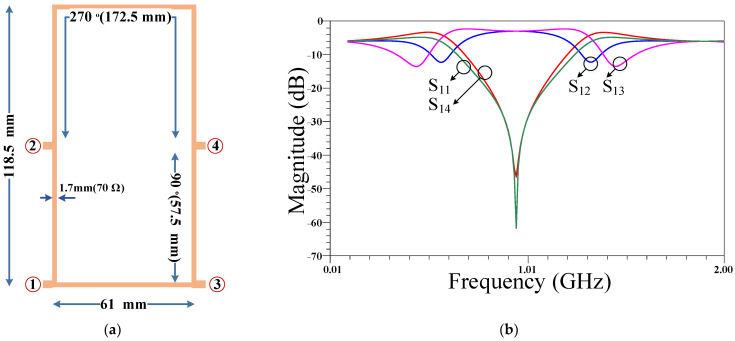
First design of the conventional RRC: (**a**) layout and (**b**) frequency response at 950 MHz.

This section presents the design steps of the proposed rat-race coupler. EM simulation in ADS (Advanced Design System) software, which is a powerful tool for designing and analyzing electromagnetic structures and devices in high frequency, is used for high-frequency simulation of the proposed design. The EM simulation method is highly accurate and can handle complex geometries, material properties, and boundary conditions. At the first step of the design procedure, the conventional rat-race coupler at the desired frequency is designed and explained. Since the desired main frequency is 950 MHz, a conventional RRC is designed and simulated using a Rogers 5880 substrate, where the obtained results are illustrated in Figure 1. As is seen, the conventional RRC suffers from extra-large size and also a lack of suppression band. Therefore, the aim of this research is to mitigate these disadvantages of the conventional RRC.

**Figure 2:**
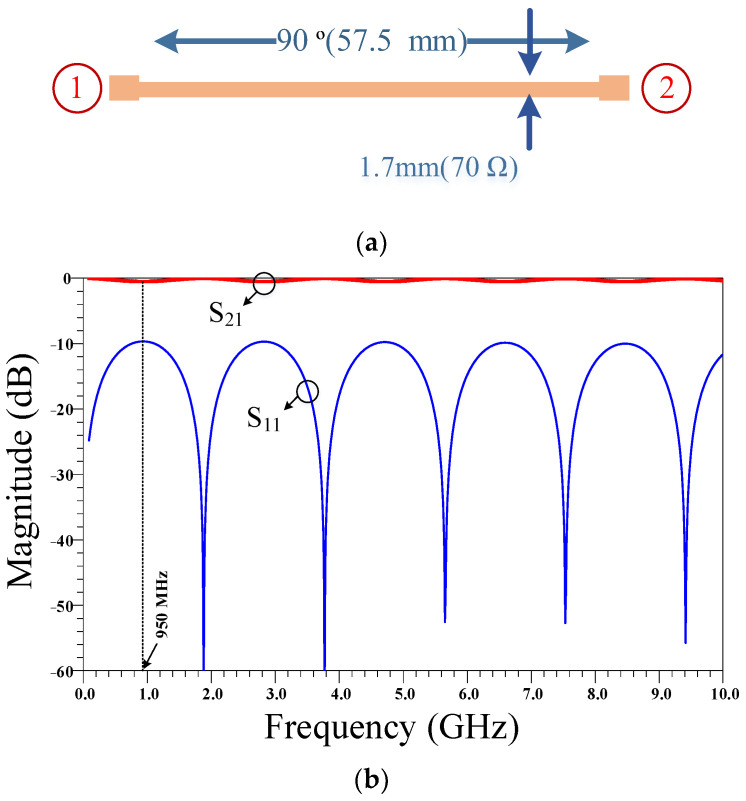
The (**a**) layout and (**b**) frequency response of a quarter wavelength (λ/4) transmission line. The physical length of this transmission line is calculated based on the operating frequency of 950 MHz and the specifications of the Rogers 5880 substrate.

The extra-large size of the conventional RRC corresponds to the large λ/4 and 3λ/4 main transmission line branches between the ports. In the next steps, two compact equivalent resonators are proposed for use instead of the conventional large transmission line branches. The layout and frequency response of the λ/4 transmission line branches are presented in Figure 2. As can be seen, in addition to the large size, there is no suppression band in the frequency response.

**Figure 3:**
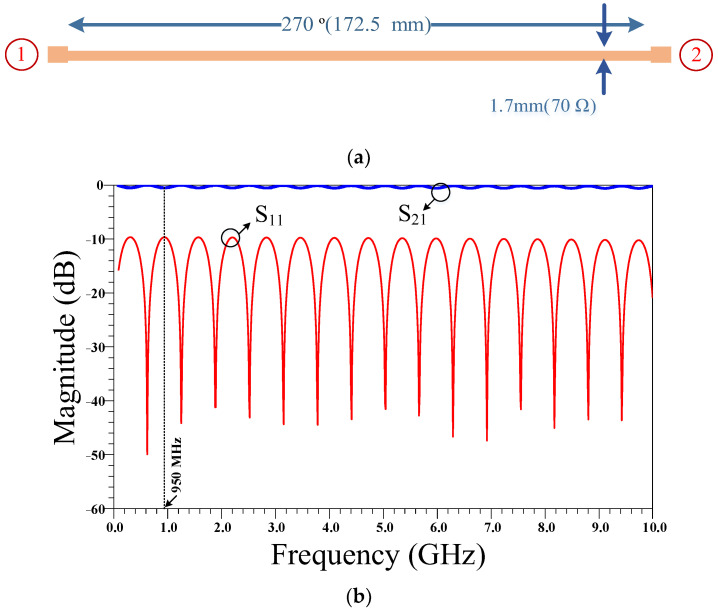
The (**a**) layout and (**b**) frequency response of a three-quarter wavelength (3λ/4) transmission line. The physical length of this transmission line is calculated based on the operating frequency of 950 MHz and specifications of Rogers 5880 substrate.

The layout and frequency response of the 3λ/4 transmission line branches are presented in Figure 3. Since the size of the 3λ/4 transmission line is so large and as there is no suppression band in the frequency response, an equivalent filter is proposed that can be used instead of the 3λ/4 transmission line branches. By using this proposed filter, the size of the RRC can be decreased and the suppression band can be obtained.

**Figure 4:**
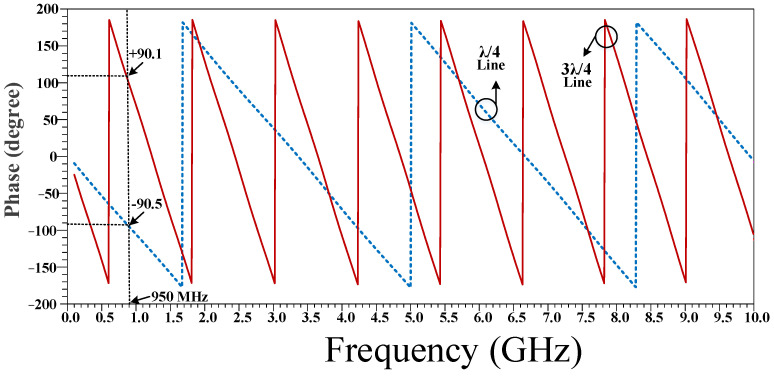
The phase characteristic of the conventional quarter wavelength (λ/4) and three-quarter wavelength (3λ/4) branches.

The phase characteristic of the conventional quarter wavelength (λ/4) and three-quarter wavelength (3λ/4) branches are shown in Figure 4. As can be seen, there is a 180 degree phase difference between the λ/4 and 3λ/4 branches.

The use of filters in microwave coupler and power divider design is an effective method for achieving compact size and harmonic suppression, in which compact filters are used instead of long conventional transmission lines. In this method, the filter and conventional line should have the same response at operating frequency. Filters are passive components that can be designed to selectively pass or reject certain frequencies depending on their configuration. One advantage of using filters in microwave coupler and power divider design is that they allow for a more compact design instead of using long conventional transmission lines, which can reduce overall size of device. This is particularly useful in applications where space is limited, such as in mobile devices or satellite communication systems. Another advantage of using filters is that they can suppress harmonics. Harmonics are unwanted frequencies that can be generated by nonlinear devices, such as amplifiers or mixers. These harmonics reduce the overall performance of the system. By using filters, these harmonics can be suppressed, leading to a cleaner output signal. Overall, the use of filters in microwave coupler and power divider design is a powerful tool for achieving compact size and harmonic suppression. By selectively passing or rejecting certain frequencies, filters can help to improve the performance of microwave systems in a variety of applications.

**Figure 5:**
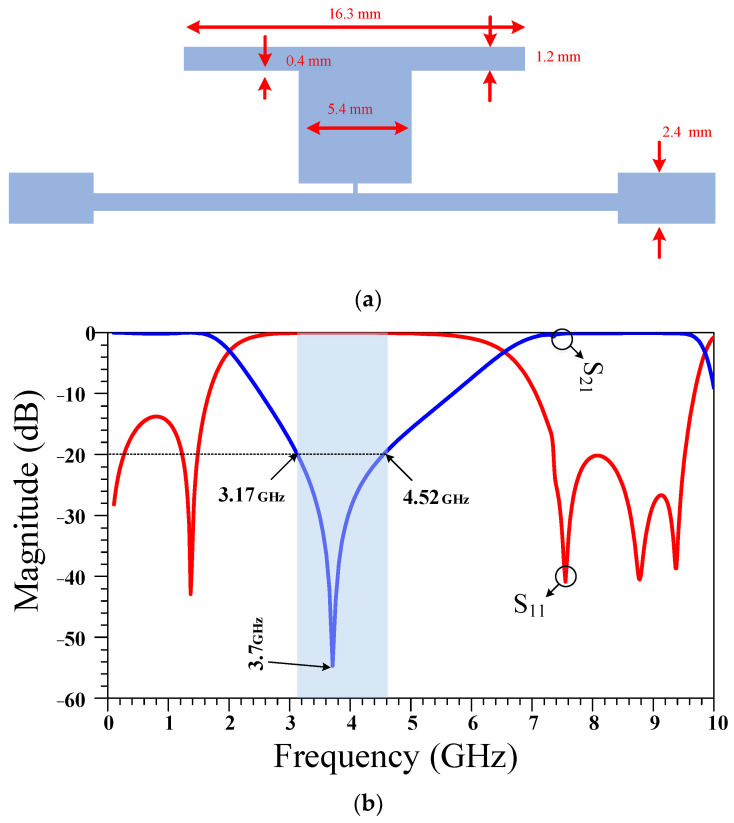
Proposed center resonator (**a**) layout, and (**b**) frequency response.

The designed proposed filter consists of two parts. These are a center resonator and two side resonators. The layout and frequency response of the proposed center resonator are depicted in Figure 5a,b, respectively. This structure creates a transmission zero at 3.7 GHz and provides a 1350 MHz suppression bandwidth with a 20 dB suppression level from 3.17 GHz to 4.52 GHz.

**Figure 6:**
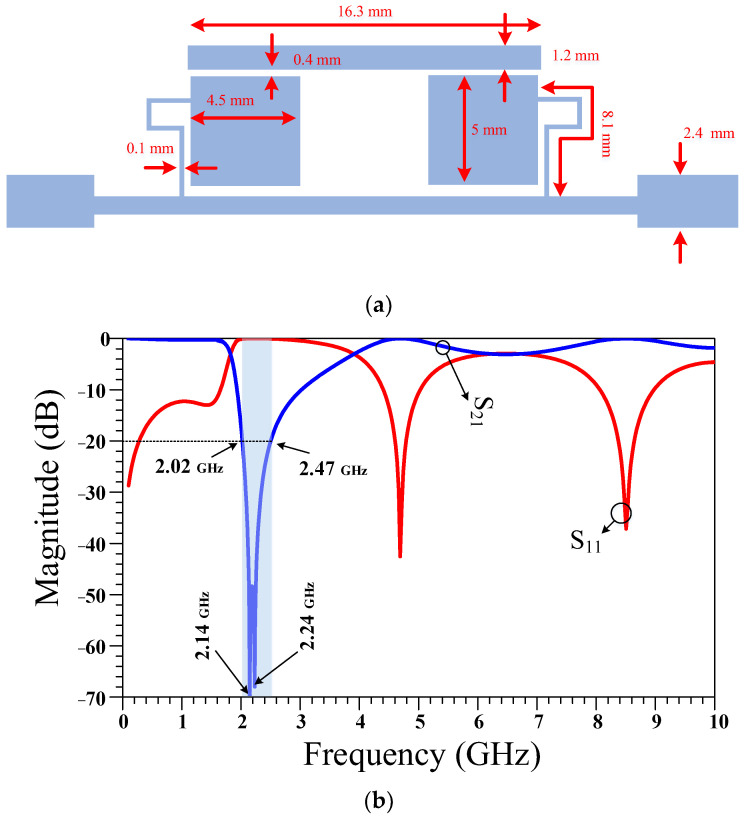
Proposed two side resonators: (**a**) layout, and (**b**) frequency response.

The layout and frequency response of the proposed two side resonators are depicted in Figure 6a,b, respectively. These resonators create two transmission zeros at 2.14 GHz and 2.24 GHz, which provides a 450 MHz suppression bandwidth with a 20 dB suppression level from 2.02 GHz to 2.47 GHz.

**Figure 7:**
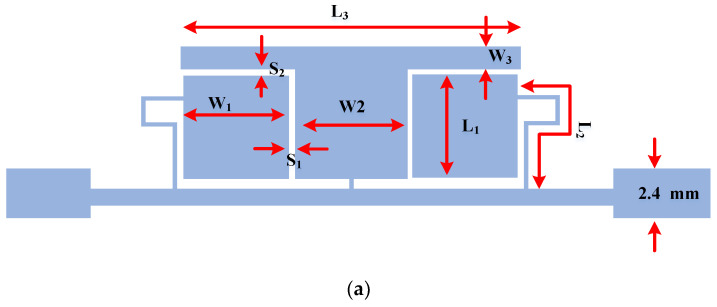

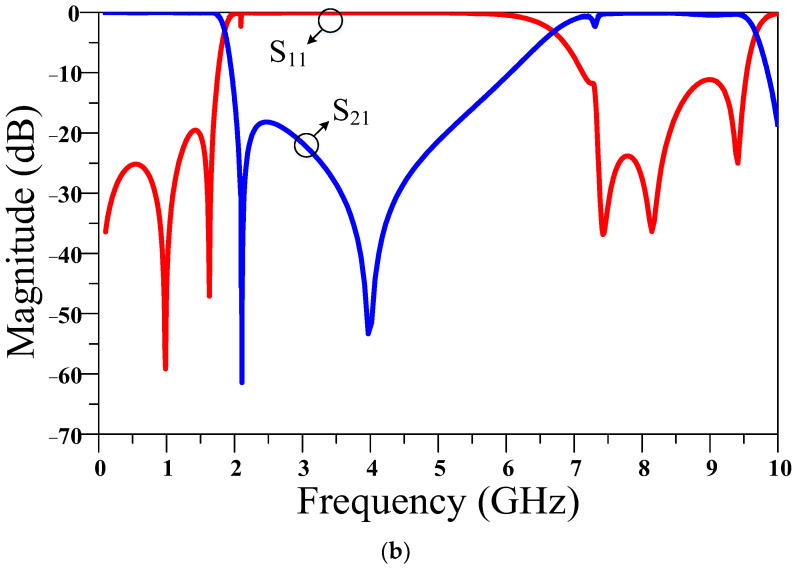
Proposed equivalent filter: (**a**) layout, and (**b**) frequency response.

By combining the proposed center resonator and two side resonators, the proposed filter can be created. The equivalent proposed filter, which is designed to be used instead of the λ/4 and 3λ/4 transmission line branches, is shown in Figure 7. The size of the deigned filter is very small, as compared with the λ/4 and 3λ/4 transmission line branches, which produces an overall size reduction for the proposed rat-race coupler.

**Figure 8:**
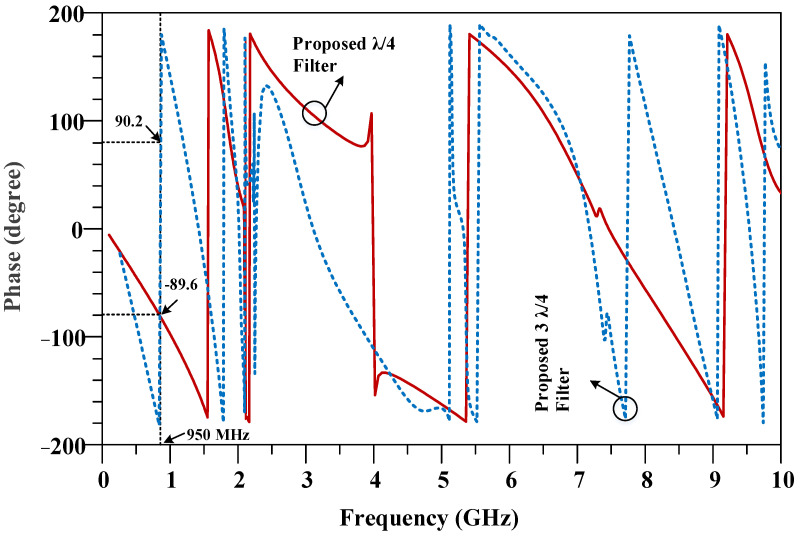
The phase characteristic of the proposed λ/4 and 3λ/4 filters.

The phase characteristics of the proposed λ/4 and 3λ/4 filters are depicted in Figure 8. As can be seen, a 180-degree phase difference between the designed λ/4 and 3λ/4 filters, which are used instead of conventional branches, is achieved. It can be concluded from comparing Figure 7b and Figure 8 with the conventional branch response, that the designed λ/4 and 3λ/4 filters have correct magnitude and phase characteristics, which demonstrates that the equivalent filters correctly work instead of conventional transmission lines at an operating frequency of 950 MHz.

## 3. The Architecture of the Proposed ANN Model

**Figure 9:**
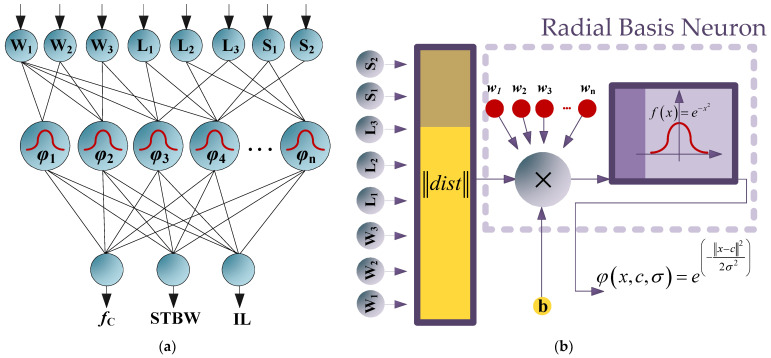
(**a**) Proposed structure of the RBF-ANN model including a single hidden layer. (**b**) Neuron model of the RBF network.

A radial basis function (RBF) is used to generate an artificial neural network (ANN). The RBF ANN is a type of artificial neural network that has become increasingly popular in recent years due to its notable capabilities in functional approximation and pattern recognition. The RBF architecture consists of three layers: input, hidden and an output layer. The hidden layer uses a radial basis function as its activation function, which allows the RBF neural network to perform non-linear transformations of the input data. The output layer is typically a linear function of the weighted inputs from the hidden layer. Figure 9 shows the proposed RBF structure for the defined artificial network and its neuron model. According to this figure, the input parameters are connected to output nodes by a single hidden layer. The filter dimensions are considered to be the input parameters for the proposed RBF model, which are W_1_, W_2_, W_3_, L_1_, L_2_, L_3_, S_1_, and S_2_, while the output parameters of the RBF model are cutoff frequency (*f*_c_), stopband bandwidth (STBW), and insertion loss (IL) parameters.

The maximum number of 70 neurons are considered in the hidden layer of the proposed ANN model. Additionally, a gradient descent-based training algorithm of Bayesian regularization backpropagation algorithm is used for the training process. The Bayesian regularization algorithm combines the advantages of regularized least-squares and backpropagation methods to train the RBF network efficiently. Applied regularization techniques can prevent the over fitting problem from occurring in the network. In addition, regularization helps to control the complexity of the RBF model and improve its generalization performance by adding regularization terms to the objective function during training. The mean-squared-error (MSE) value is used to quantify the average squared difference between the network predicted outputs and the target outputs during the training process. Lower MSE values indicate a better training performance and the training goal is considered as zero in the proposed model, which is the desired value for the performance metric of MSE. The Gaussian activation function is utilized for the hidden layer in the proposed model. The spread (width) of the radial basis functions in the hidden layer is considered to be equal to 1, which determines the influence of each radial basis function on the network output. The performance step size and the transfer function step size, which are considered during performance gradient calculation, are equal to 0.001 and 0.01, respectively. The ‘newrb’ function in the Neural Network Toolbox in MATLAB software is used to design the presented RBF ANN model.

The analysis of the presented RBF network can be written as shown in Equations (1)–(3) [43,44]. The Gaussian function, which is used as the radial basis function in the RBF network, is written in Equation (1).

(1) $$\varphi \left(x,c,\sigma \right)=e^{\left(-\frac{{\left\|x-c\right\|}^{2}}{2\sigma^{2}}\right)},$$

where ‘*x*’ is the input vector, ‘*c*’ is the center of radial basis function, and ‘σ*’* is the width of the radial basis function. By applying the radial basis function to the input pattern and the center of the neuron, the activation of a neuron in the RBF can be computed, which is written in Equation (2).

(2) $$a_{i}=\varphi \left(x,c_{i},\sigma_{i}\right)$$

In this equation, ‘*x*’ is the input vector. Additionally, ‘*c_i_*’ and ‘*σ_i_*’ are the center and width of the *i*th neuron. Finally, the output of the RBF network can be calculated via a weighted summation of the activation functions from the hidden layer, which is written in Equation (3). In this equation, ‘*w_i_*’ is the weight of the connection between the *i*th neuron in the hidden layer and the output layer.

(3) $$y=\sum_{i=1}^{n}w_{i}a_{i}$$

### Results of the Proposed ANN Model

**Figure 10:**
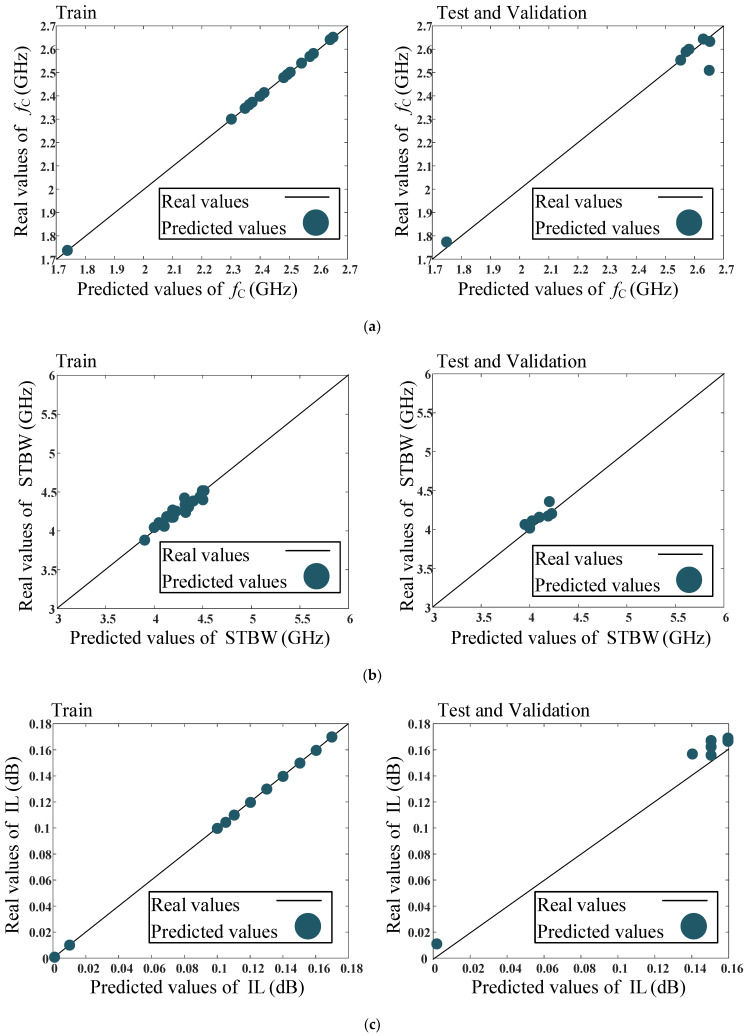
Comparison between the real and predicted values for the training and testing data using the proposed model: (**a**) *f*_c_ (GHz), (**b**) STBW (MHz) and (**c**) IL (dB). The real values are shown as solid lines. Real values are extracted values from EM-simulation of the filter, which are used to train and test the proposed model, while the predicted values are the parameters, which are predicted by the trained network.

The real and predicted comparison values of cutoff frequency (*f*_c_), stopband bandwidth (STBW), and insertion loss (IL) parameters for training and testing data are shown in Figure 10. As can be seen, the predicted data are obtained accurately. Ideally, the values predicted by the proposed model should be aligned closely with the real values. If the predicted values match the real values closely and follow the general trend of the data, the predicted dots will be located on the Y = X solid line, which suggests that the ANN model is a good fit for the data. On the other hand, if there are substantial differences between the predicted and real values, the predicted dots will be some distance from the Y = X solid line, which may indicate that the model is not capturing the relationship adequately or that there are other factors influencing the dependent variable that are not accounted for in the model. According to Figure 10, the predicted values are located approximately on the Y = X solid line for both training and testing processes, which indicates the precision and validity of the proposed ANN model.

**Figure 11:**
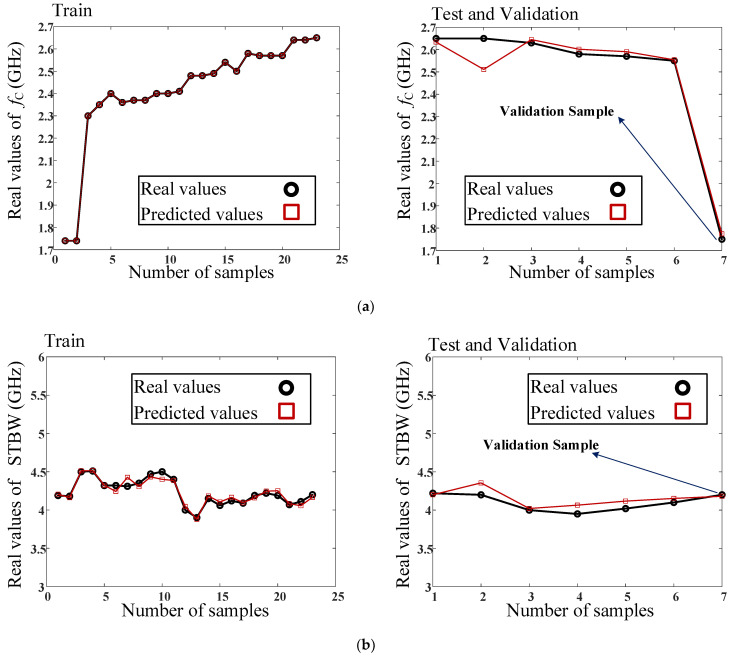

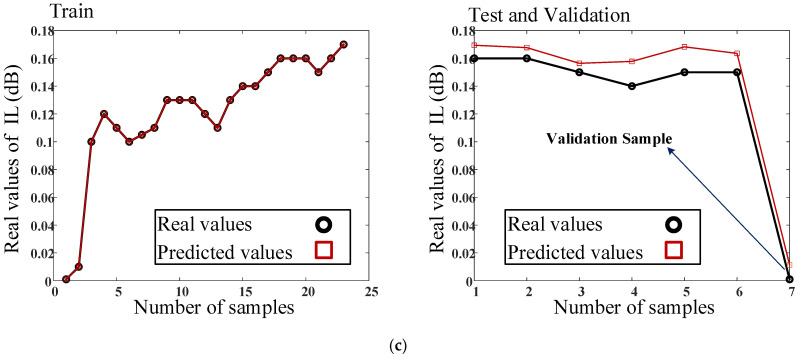
Real and predicted testing and training values of (**a**) *f*_c_ (GHZ), (**b**) STBW (MHz), and (**c**) IL (dB) parameters versus number of data samples in the proposed model. The real values are shown as solid line. Real values are extracted values from EM-simulation of the filter. These are used to train and test the proposed model, while the predicted values are the parameters, which are predicted by the trained network.

Real and predicted testing and training values of *f*_c_ (GHz), STBW (MHz), and IL (dB) parameters versus number of data samples in the proposed model are shown in Figure 11. As is seen, the circuit parameters are predicted accurately. Two samples are chosen for validation, and these are shown in Figure 11. The validation samples validate the accuracy of the proposed model and help to design the resonator with the desired parameters.

The real data of the training, testing, and validation procedures, obtained using the proposed model, are listed in Table 1. The final results of the proposed ANN model are listed in Table 2, which show high accuracy of the prediction for proposed model. The errors, which are reported in this table, correspond to the denormalized data. According to this table, the model is trained perfectly using the training data. The validation data are extracted from the proposed ANN model and are then used to design the equivalent filter for the proposed RRC with desirable parameters. As is seen in Table 1, the eight parameters of the filter dimensions are considered as the input parameters of the model and the three output parameters of the model are related to the results of the filter frequency response. Additionally, 23 samples and 6 samples are considered for the training and testing of the proposed model, respectively. Additionally, the validation sample verifies the results of the proposed model. Finally, the validation sample is simulated and fabricated, which demonstrates the validity of the predicted results expressed by the proposed ANN.

## 4. Results

**Figure 12:**
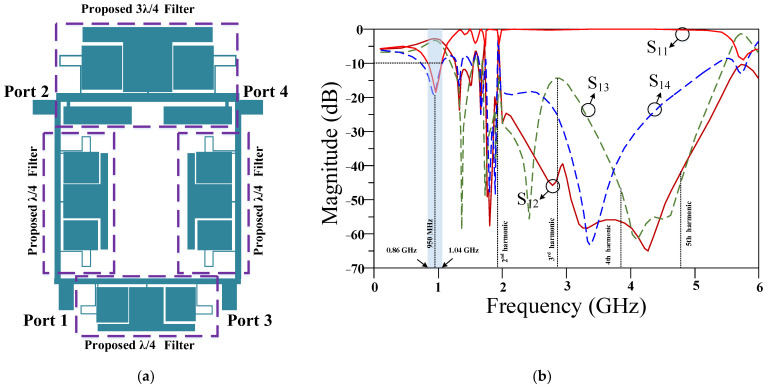
Proposed Rate Race Coupler: (**a**) layout and (**b**) frequency response.

By substituting the proposed filter instead of the large λ/4 and 3λ/4 main transmission line branches, the final RRC is achieved. The layout and frequency response of the proposed RRC is depicted in Figure 12. The obtained size of the proposed RRC is 0.20 λ × 0.12 λ, or 46.5 mm × 27.5 mm. This shows a size reduction greater than 82% size reduction in comparison to the conventional RRC.

The EM simulations response of the proposed RRC is depicted in Figure 12b. The EM simulation results show that the proposed RRC has desirable performance at an operating frequency of 950 MHz, with 180 MHz bandwidth. The insertion loss at 950 MHz is 0.01 dB and return loss is better than 19 dB. Additionally, the proposed RRC has a good performance at higher frequencies and both S_21_ and S_31_ curves have low values at harmonic frequencies. The harmonic suppression levels for 2nd, 3rd, 4th and 5th harmonics are more than 20 dB, 15 dB, 48 dB and 42 dB, respectively, which are the worst reported values for two curves of S_21_ and S_31._

**Figure 13:**
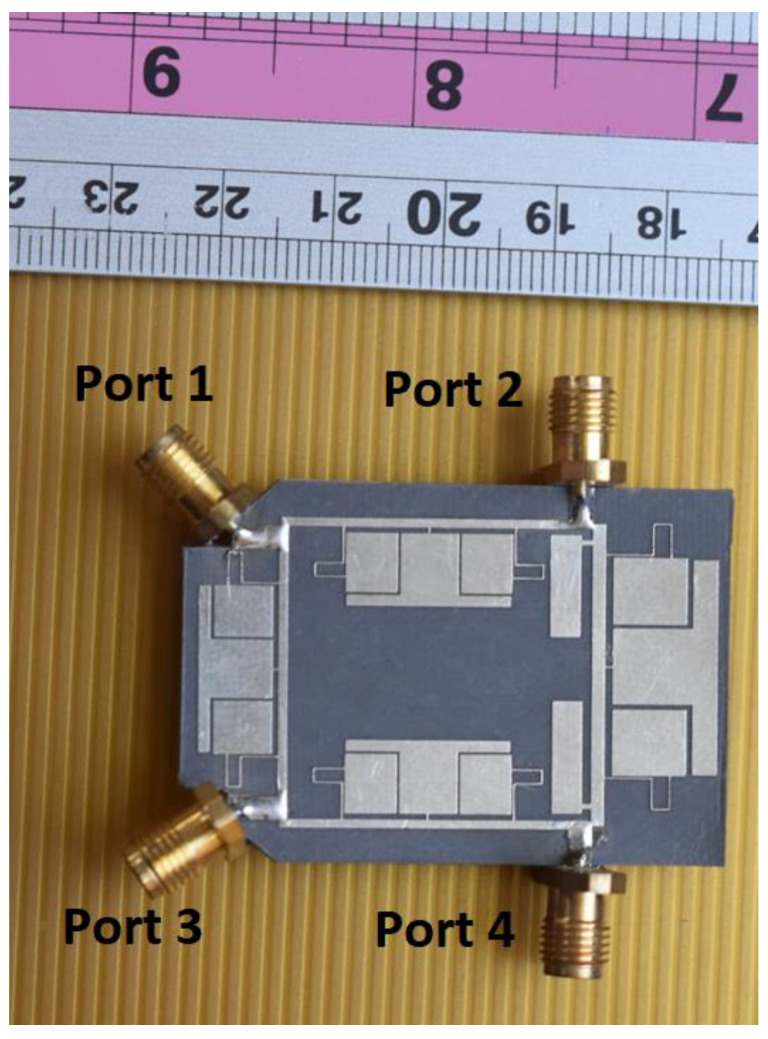
Fabricated prototype of the proposed Rate Race Coupler.

**Figure 14:**
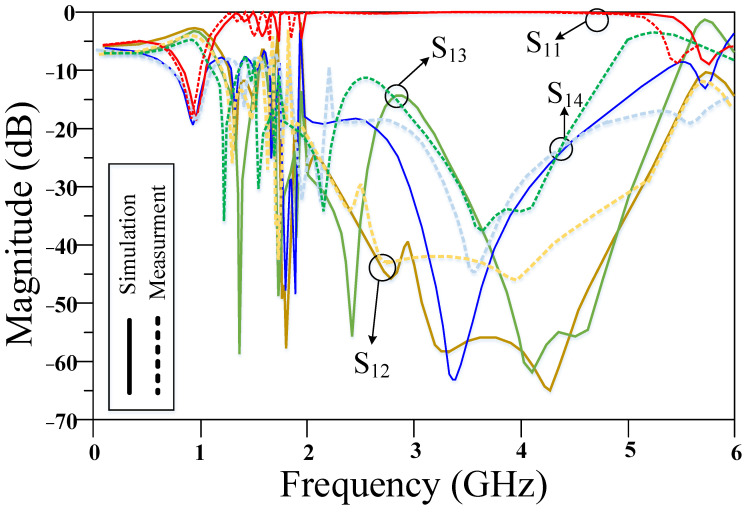
Measured results of the proposed RRC.

The proposed RRC is fabricated on the Rogers 5880 substrate, which is shown in Figure 13. The measured results of the designed RRC are illustrated in Figure 14. As seen, the designed device works correctly at 950 MHz with 180 MHz bandwidth, which shows FBW of 19%. Additionally, the obtained magnitude of S_11_, S_21_, S_31_, and S_41_ at the operating frequency are −18 dB, −3.15 dB, −3.2 dB, and −19 dB, respectively. Four harmonics are suppressed in the proposed device with the suppression levels of, respectively, 23 dB, 15 dB, 46 dB, 42 dB. It can be concluded from Figure 14 that a wide suppression band is achieved for the proposed coupler. Conversely, the fabricated device correctly works at the operating bandwidth with desired performance, while it provides harmonic rejection in higher frequencies with a high attenuation level.

**Figure 15:**
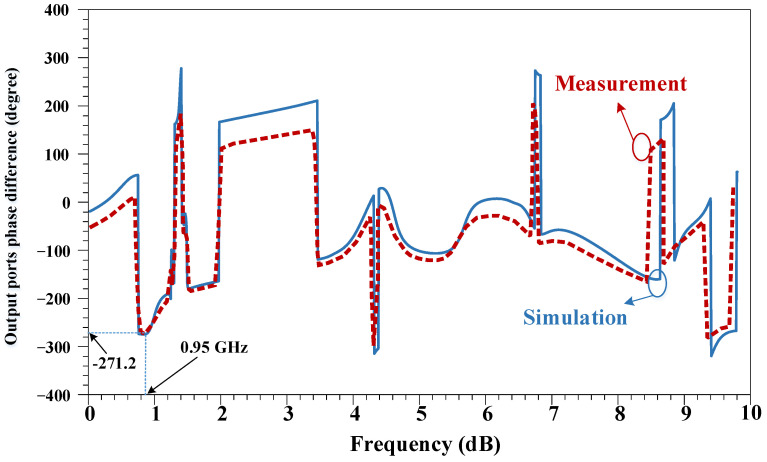
The output phase difference of the proposed RRC device.

The output phase difference of the proposed RRC device is shown in Figure 15. This phase difference corresponds to the phase difference of the output ports in the designed coupler. As is seen, the experimental values of −271.2° are obtained at the operating frequency, which shows the high phase accuracy of the proposed coupler.

The performance of the proposed coupler is compared with the other related works in Table 3. As can be seen in the table, the proposed RRC has the best performance, compared with the related works, in the terms of harmonic suppression and sire reduction simultaneously.

## 5. Conclusions

In this paper, the design of a proposed RRC with harmonic suppression and size reduction is described and presented by using an RBF-ANN. An RBF network is used in this design to predict the desired parameters of the proposed RRC. The performance of the proposed coupler is analyzed using a full-wave electromagnetic simulator, and the simulation results are presented and discussed. Finally, the RRC is fabricated using a Rogers 5880 and measured using a vector network analyzer (VNA). The experimental results demonstrate that the proposed coupler exhibits excellent performance characteristics, including a high isolation of 19 dB, low insertion loss of 0.2 dB, a size reduction of more than 82%, and the suppression of the 2nd to 5th harmonics. The proposed design is highly compact, making it suitable for integration into various microwave circuits. The experimental results confirm the effectiveness of the proposed design, which can be used as a fundamental building block for many microwave circuits.

## Figures and Tables

**Table 1:** Real data of train, test, and verification procedures, using the proposed model.

Training Values and Design Parameters														
Input Parameters									Real Output Param.			Predicted Output Param.		
	W_1_ (mm)	W_2_ (mm)	W_3_ (mm)	L_1_ (mm)	L_2_ (mm)	L_3_ (mm)	S_1_ (mm)	S_2_ (mm)	*f*_c_ (GHz)	STBW (MHz)	IL (dB)	*f*_c_ (GHz)	STBW (MHz)	IL (dB)
1	5	5.4	1.3	5	8.1	16.3	0.3	0.4	1.74	4.19	0.01	1.740	4.206	0.001
2	5	5.2	1.3	5	8.1	16.3	0.3	0.5	1.74	4.18	0.01	1.740	4.162	0.010
3	4	4.2	0.8	4	6.7	12.9	0.3	0.3	2.3	4.5	0.1	2.300	4.513	0.100
4	4	4.2	0.7	4	6.7	12.9	0.2	0.3	2.35	4.51	0.12	2.350	4.514	0.120
5	4	4.2	0.7	3.8	6.7	12.9	0.2	0.4	2.4	4.32	0.11	2.400	4.323	0.110
6	4	4.2	0.8	3.8	6.7	12.9	0.2	0.4	2.36	4.32	0.1	2.360	4.242	0.100
7	4	4.2	0.9	3.8	6.7	12.9	0.2	0.3	2.37	4.31	0.11	2.370	4.427	0.105
8	4	4	0.8	3.8	6.7	12.9	0.3	0.4	2.37	4.35	0.11	2.370	4.306	0.110
9	3.8	4	0.8	3.8	6.7	12	0.2	0.3	2.4	4.47	0.13	2.400	4.432	0.130
10	3.8	4	0.9	3.8	6.7	12	0.2	0.3	2.4	4.5	0.13	2.400	4.401	0.130
11	3.8	3.8	0.9	3.8	6.7	12	0.3	0.3	2.41	4.4	0.13	2.410	4.385	0.130
12	3.8	3.8	0.9	3.8	6.7	11.8	0.2	0.4	2.48	4	0.12	2.480	4.046	0.120
13	3.8	3.8	1	3.8	6.7	11.8	0.2	0.4	2.48	3.9	0.11	2.480	3.879	0.110
14	3.8	3.8	0.8	3.8	6.7	11.8	0.2	0.4	2.49	4.15	0.13	2.490	4.182	0.130
15	3.6	3.8	0.8	3.8	6.7	11.8	0.4	0.4	2.54	4.06	0.14	2.540	4.104	0.140
16	3.6	3.8	0.8	3.8	6.7	11.6	0.3	0.4	2.5	4.12	0.14	2.500	4.165	0.140
17	3.6	3.8	0.8	3.8	6.5	11.6	0.3	0.4	2.58	4.09	0.15	2.580	4.095	0.150
18	3.6	3.8	0.7	3.8	6.5	11.6	0.3	0.4	2.57	4.19	0.16	2.570	4.158	0.160
19	3.6	3.8	0.7	3.8	6.5	11.6	0.3	0.3	2.57	4.22	0.16	2.570	4.246	0.160
20	3.6	4	0.7	3.8	6.5	11.6	0.2	0.3	2.57	4.19	0.16	2.570	4.252	0.160
21	3.4	4	0.8	3.8	6.5	11.6	0.4	0.3	2.64	4.07	0.15	2.640	4.081	0.150
22	3.4	4	0.7	3.8	6.5	11.6	0.4	0.3	2.64	4.11	0.16	2.640	4.055	0.160
23	3.4	4	0.7	3.8	6.5	11.2	0.2	0.3	2.65	4.2	0.17	2.650	4.166	0.170
**Testing Values**														
**Input Parameters**									**Real Output Param.**			**Predicted Output Param.**		
	W_1_ (mm)	W_2_ (mm)	W_3_ (mm)	L_1_ (mm)	L_2_ (mm)	L_3_ (mm)	S_1_ (mm)	S_2_ (mm)	*f*_c_ (GHz)	STBW (MHz)	IL (dB)	*f*_c_ (GHz)	STBW (MHz)	IL (dB)
1	3.4	4	0.8	3.8	6.5	11.2	0.2	0.3	2.65	4.22	0.16	2.633	4.200	0.169
2	3.4	4	0.8	3.8	6.5	11.2	0.2	0.2	2.65	4.2	0.16	2.511	4.355	0.167
3	3.4	3.8	0.8	3.8	6.5	11.2	0.3	0.4	2.63	4	0.15	2.644	4.021	0.156
4	3.4	3.8	0.8	4	6.5	11.2	0.3	0.4	2.58	3.95	0.14	2.601	4.064	0.157
5	3.4	3.8	0.7	4	6.5	11.2	0.3	0.4	2.57	4.02	0.15	2.590	4.117	0.168
6	3.4	3.8	0.7	4	6.6	11.2	0.3	0.4	2.55	4.1	0.15	2.554	4.152	0.163
**Validation Values**														
**Input Parameters**									**Real Output Param.**			**Predicted Output Param.**		
	W_1_ (mm)	W_2_ (mm)	W_3_ (mm)	L_1_ (mm)	L_2_ (mm)	L_3_ (mm)	S_1_ (mm)	S_2_ (mm)	*f*_c_ (GHz)	STBW (MHz)	IL (dB)	*f*_c_ (GHz)	STBW (MHz)	IL (dB)
1	5	5.4	1.2	5	8.1	16.3	0.4	0.4	1.75	4.2	0.01	1.774	4.180	0.011

**Table 2:** Final results of the proposed ANN model.

	*f*_c_ (GHz) Errors			STBW (MHz) Errors			IL (dB) Errors		
	Train	Test	Valid.	Train	Test	Valid.	Train	Test	Valid.
MRE	2.45 × 10^−15^	0.0137	0.0139	0.0088	0.0189	0.0046	7.48 × 10^−14^	0.0816	10.3425
RMSE	6.78 × 10^−15^	0.0587	0.0243	0.0471	0.0918	0.0191	6.64 × 10^−16^	0.0131	0.0103

**Table 3:** Performance comparison of the proposed RRC and the related works.

Ref.	Size Reduction%	No. of Suppressed Harmonic	Return Loss	Isolation
[45]	94	None	16	35
[37]	87	None	20	21
[36]	57	None	15	43
[38]	85	9	45	50
[39]	54	1	20	20
[41]	55	2	22	20
[42]	None	1	18	20
Proposed RRC	82	4	18	19

## Data Availability

All the material conducted in the study is mentioned in article.
